# Reward Processing in Trichotillomania and Skin Picking Disorder

**DOI:** 10.1007/s11682-021-00533-5

**Published:** 2021-08-19

**Authors:** Jon E. Grant, Tara S. Peris, Emily J. Ricketts, Richard A.I. Bethlehem, Samuel R. Chamberlain, Joseph O’Neill, Jeremiah M. Scharf, Darin D. Dougherty, Thilo Deckersbach, Douglas W. Woods, John Piacentini, Nancy J. Keuthen

**Affiliations:** a Department of Psychiatry & Behavioral Neuroscience University of Chicago, Chicago, IL, USA; b Department of Psychiatry and Biobehavioral Sciences, University of California, Los Angeles, CA, USA; c Department of Psychiatry, University of Cambridge, UK; d Department of Psychiatry, Faculty of Medicine, University of Southampton, UK; and Southern Health NHS Foundation Trust, UK; e Department of Psychiatry, Massachusetts General Hospital and Harvard Medical School, Boston, USA; f Department of Psychology, Marquette University, Milwaukee, WI, USA

**Keywords:** trichotillomania, skin picking disorder, imaging, reward, fMRI, neurobiology

## Abstract

**Background:**

Trichotillomania (hair pulling disorder) and skin picking disorder are common and often debilitating mental health conditions, grouped under the umbrella term of body focused repetitive behaviors (BFRBs). Although the pathophysiology of BFRBs is incompletely understood, reward processing dysfunction has been implicated in the etiology and sustention of these disorders. The purpose of this study was to probe reward processing in BFRBs.

**Methods:**

159 adults (125 with a BFRB [83.2% (n=104) female] and 34 healthy controls [73.5% (n=25) female]) were recruited from the community for a multi-center between-group comparison using a functional imaging (fMRI) monetary reward task. Differences in brain activation during reward anticipation and punishment anticipation were compared between BFRB patients and controls, with stringent correction for multiple comparisons. All group level analyses controlled for age, sex and scanning site.

**Results:**

Compared to controls, BFRB participants showed marked hyperactivation of the bilateral inferior frontal gyrus (pars opercularis and pars triangularis) compared to controls. In addition, BFRB participants exhibited increased activation in multiple areas during the anticipation of loss (right fusiform gyrus, parahippocampal gyrus, cerebellum, right inferior parietal lobule; left inferior frontal gyrus). There were no significant differences in the win-lose contrast between the two groups.

**Conclusions:**

These data indicate the existence of dysregulated reward circuitry in BFRBs. The identified pathophysiology of reward dysfunction may be useful to tailor future treatments.

## Introduction

Trichotillomania (TTM; also known as Hair Pulling Disorder) and Skin Picking Disorder (SPD), are characterized by repeated pulling out of hair resulting in hair loss or picking at skin resulting in tissue damage, respectively. These disorders have been conceptualized under the larger umbrella of body focused repetitive behavior disorders (BFRBs) and often result in significant psychosocial impairment (Tucker et al., 2011). Although both psychosocial and psychopharmacological treatments have demonstrated some degree of efficacy for BFRBs, many people fail to respond or exhibit only partial responses to these interventions (Sani et al., 2019).

One issue that has hampered treatment development to date, and the formulation of brain-based disease models, is that the understanding of the neurobiology of BFRBs remains limited. BFRB neuroimaging research comparing patients to matched controls has identified, in small, single-site samples (Ns ranging from 10-76) of participants, evidence of abnormalities in several regions of the brain which have included areas involved in habit formation (e.g., dorsal striatal areas), emotional regulation (e.g., amygdala and hippocampal areas), memory processing (e.g., temporal lobe), self-monitoring and awareness (e.g., precuneus), reward processing (e.g., ventral striatum, frontal hemisphere, bilateral cuneus), visual processing of disgust (e.g., insula and putamen), and generation and suppression of motor responses (e.g., inferior frontal gyrus) (Swedo et al., 1991; Grachev 1997; O’Sullivan et al., 1997; Stein et al., 1997; Rauch et al., 2007; Keuthen et al., 2007; Chamberlain et al., 2008, 2010; 2018; Lee et al., 2010; grant et al., 2013; Roos et al., 2015; Odlaug et al., 2016; Chienle et al., 2018; Isobe et al., 2018; Wabnegger and Schienle 2019). With such small samples and conflicting findings, if treatments are to target pathophysiology of BFRBs there remains a substantive need for further work aimed at understanding more precisely the neurobiological underpinnings of BFRBs.

One potential promising area for exploration is reward processing, based on several convergent lines of thinking. The pulling and picking of BFRBs is often described as pleasurable (Arzeno et al., 2006) and people will report urges to engage in the behavior that mirror those described by people with substance use problems; and that undertaking the behavior leads to transient reduction or relief of the urge, though this is short-lived (Grant et al., 2007). Additionally, early data suggested that people with BFRBs were more likely than controls to have first-degree relatives with substance addictions, disorders typically characterized by reward processing abnormalities (Schlosser et al., 1994). Although limited in number, available double-blind placebo-controlled treatment trials that have shown some benefit for BFRBs have used pharmacological agents modulating glutamate and dopamine (Deepmala et al., 2015), both of which seem integral to reward processing. In a previous study examining reward circuitry activation, White and colleagues (2013) examined 13 adults with TTM (unmedicated and compared to 12 controls) using fMRI with a monetary incentive delay (MID) task. In a region-of-interest analysis, TTM patients exhibited decreased nucleus accumbens (NAcc) activation for gain anticipation and increased NAcc activation for gain and loss outcomes, versus controls. However, these findings were not significant at the whole brain level. At the level of the whole brain, loss anticipation showed less activation of the left putamen and insula in TTM than controls.

Given this background, there is some suggestion that TTM and SPD may represent problems of disordered reward processing, but further testing of this hypothesis via neuroimaging is needed. Therefore, a greater understanding of reward processing should allow for improved prevention and treatment strategies. Thus, the objective of this study was to examine reward circuitry activation in a large multi-site sample of patients with BFRBs compared to controls.

## Methods

Participants included 159 adults recruited from the community as having either a BFRB (trichotillomania, skin picking disorder, or both as their primary psychiatric problem) or being a healthy control. Three sites were involved in recruitment: University of Chicago, University of California, Los Angeles, and Massachusetts General Hospital/Harvard Medical School.

Inclusion criteria for the clinical sample was: a) DSM-5 diagnosis of trichotillomania and/or skin picking disorder as the primary psychiatric conditions; b) aged 18 to 65; c) fluency in English; and d) capable of providing informed consent/assent. Inclusion criteria for the healthy controls were the same except they could have no current or lifetime history of any DSM-5 psychiatric disorder. BFRB participants could have comorbid psychiatric disorders (based on the Mini International Neuropsychiatric Interview 7.0; (Sheehan et al., 1998) as long as TTM or SPD were the primary psychiatric condition.

Exclusion criteria for the clinical sample and healthy controls were: (a) current or lifetime diagnosis of any serious medical or psychiatric illness (including substance use disorder) that would preclude successful study participation; (b) neurological conditions that would preclude completion of neurocognitive tasks; (c) use of psychotropic medications unless the dose had been stable for at least the past 3 months; (d) body metal other than dental fillings (assessed using a neuroimaging screening form); (e) positive pregnancy test; and (f) medical condition or other factor contraindicating neuroimaging.

### Procedures

Potential participants were screened by the study site coordinator. Prior to obtaining written informed consent, the investigators provided a complete description of the study, discussed potential risks, and answered questions regarding the study. After that, participants provided written informed consent. Participants received up to $200 for participation as reimbursement for their time.

All participants underwent a diagnostic interview and were asked to complete an “MR Screening Form” to rule out any conditions that preclude MR scanning.

#### MRI Neuroimaging

We used a multi-site neuroimaging design involving equal numbers of participants across three sites: (1) in the Massachusetts General Hospital Martinos Center for Biomedical Imaging, (2) the Staglin Center for Cognitive Neuroscience at the UCLA Semel Institute for Neuroscience and Human Behavior, and (3) the Department of Psychiatry and Behavioral Neuroscience at the University of Chicago. As described above, participants were screened for scanner compatibility at the outset, and we scanned eligible participants sequentially. Imaging was performed on a 3-Tesla MRI scanner at all three sites with all scanners synchronized. Each MRI scanning session lasted no more than 75 minutes. Task order was pseudo-randomized and counterbalanced across subjects. All tasks were presented using Eprime software. Participants were instructed in the behavioral tasks that they would engage in while in the fMRI scanner. The participants were given headphones, ear protectors, and a head restraining device was used to reduce excess motion. We first acquired high resolution, anatomical images, typically about 15 minutes, before undertaking the fMRI sequence.

#### fMRI Task: Monetary Reward Task

The monetary reward task was used to examine reward processing (Knutson et al., 2001). The version of the task deployed enabled separate analysis of neural activation for anticipated monetary reward, and for anticipated monetary loss, on the task. The task is comprised of 72 trials, and each trial lasts approximately 6 seconds (range 3–10 sec). A cue was presented for 500 milliseconds and signaled a potentially rewarding (a circle) or non-rewarding (a square) trial. Participants were instructed that the game’s goal is to see how much money they can win. If they saw a circle with one line through it, it meant they could win $1. If they saw a square with one line through it, it meant they could lose $1 if they did not press the button at the correct time. If they saw a circle with two lines through it, they could win $5. If they saw a square with two lines through it, they could lose $5. Participants were given feedback (reward or no reward). Each participant underwent 10 practice trials, and based on these practice trials, each individual had a time limit to respond to the target during the task (based on their shortest reaction time during the practice sessions). By increasing and decreasing the time limit, participants were rewarded in 50% of the reward trials. The design of the task allows for the modeling of response to anticipation of reward or loss to be independent from that of actual reward or loss.

#### Image acquisition

Imaging was acquired across three scanning sites (University of Chicago, University of California, Los Angeles, and Massachusetts General Hospital/Harvard Medical School) using a unified acquisition protocol. Structural scans were acquired on a 2 Siemens Magnetom Prismafit 3T scanners (UCLA and MGH/Harvard) and one Philips Achieva 3T MRI scanner with dStream (Chicago) all with 32-channel head coils, using a MPRAGE acquisition sequence with the following parameters: slab orientation = sagittal, FOV 256x256x176, voxel size 1x1x1 mm3, inversion delay time TI = 900 ms, TR = 2310 ms, TE = 2.9 ms flip angle = 9 degree. Functional task MRI was acquired with a single-shot gradient echo planar imaging sequence. Thirty-nine interleaved axial slices parallel to the AC–PC line covering the whole brain were acquired: TR = 2000 m s, TE = 28 ms, flip angle = 90°, field of view = 210 × 210 mm, matrix = 205 × 205, voxels size 3.2x3.2x3.1mm and a 20% distance factor, GRAPPA acceleration factor = 2 (for the 2 Siemens scanners) and SENSE acceleration factor 2 (for the Philips scanner).

#### Image processing

First level fMRI data processing was performed using FEAT (FMRI Expert Analysis Tool) Version 6.00 (FMRIB’s Software Library, www.fmrib.ox.ac.uk/fsl). Registration to high resolution structural and/or standard space images was done using FLIRT (Jenkinson et al., 2002). Registration from high resolution structural to standard space was refined using FNIRT nonlinear registration (Andersson et al., 2007). The following pre-statistics processing was applied; motion correction using MCFLIRT (Jenkinson et al., 2002); slice-timing correction using Fourier-space time-series phase-shifting; non-brain removal using BET (Smith 2002); spatial smoothing using a Gaussian kernel of FWHM 5mm; grand-mean intensity normalization of the entire 4D dataset by a single multiplicative factor; highpass temporal filtering (Gaussian-weighted least-squares straight line fitting, with sigma=50.0s). Time-series statistical analysis was done using FILM with local autocorrelation correction fitting contrasts for anticipation in win and lose trials as well as the contrast between them. All first level outputs were manually inspected for potential registration errors.

Contrasts from first level analysis were entered into higher level analyses carried out using FEAT (FMRI Expert Analysis Tool) Version 6.00 (FMRIB’s Software Library, www.fmrib.ox.ac.uk/fsl). Z (Gaussianised T/F) statistic images were thresholded non-parametrically using clusters determined by Z>2.3 corresponding to a p-value of 0.01 and a (corrected) cluster significance threshold of P=0.05. All group level analyses included age, sex and scanning site as potential confounding factors. Rendered thresholded z-maps were subsequently visualized using the glass-brain plotting tool and cope estimates using ggstatsplot.

Following analysis of main effects, we extracted cope estimates for post-hoc analysis within condition and picker and puller sub-groups from within main effect thresholded regions of interest using Featquery to extract cope estimates for peak coordinates. All post-hoc analysis of estimates were conducted using non-parametric Kruskal Wallis one-way analysis of variance for main effect and a Dwass-Steel-Crichtlow-Fligner test for pairwise comparisons. All pairwise comparisons were corrected for multiple comparisons using Benjamini and Hochberg corrections (Benjamini and Hochberg 1995). For visualization we also included individual cope estimates of main effects as assessed using the Bayesian analog of a students t-test.

## Results

### Sample Characteristics

The sample included 159 participants (125 adults with a BFRB and 34 adults as healthy controls), of which 49 had TTM, 51 had SPD, and 25 had both TTM and SPD. Of the 125 adults with BFRBs, 83.2% (n=104) were female, the mean age was 29.1 (9.2), and the mean age of BFRB onset was 13.1 (6.4) years. Of the 34 healthy controls, 73.5% (n=25) were female and mean age was 26.8 (7.6) years. Groups did not differ in terms of gender or age as assessed using a Wilcoxon signed rank test.

### Behavior

BFRB participants did not significantly differ from controls in reaction times [*Win anticipation*; 391 vs 377 msec *T*(68.81)=1.40, *p* =.*17*. *Loss anticipation;* 389 vs 375 msec *T*(71.50)=1.57, *p* =.*27*]. Reaction times did not differ between TTM, SPD, and comorbid participants [*Win anticipation*; *F*(3,78.11)=1.64, *p*=.19. *Loss anticipation; F*(3,76.83)=1.04, *p*=.38].

### Neuroimaging

**Figure 1:**
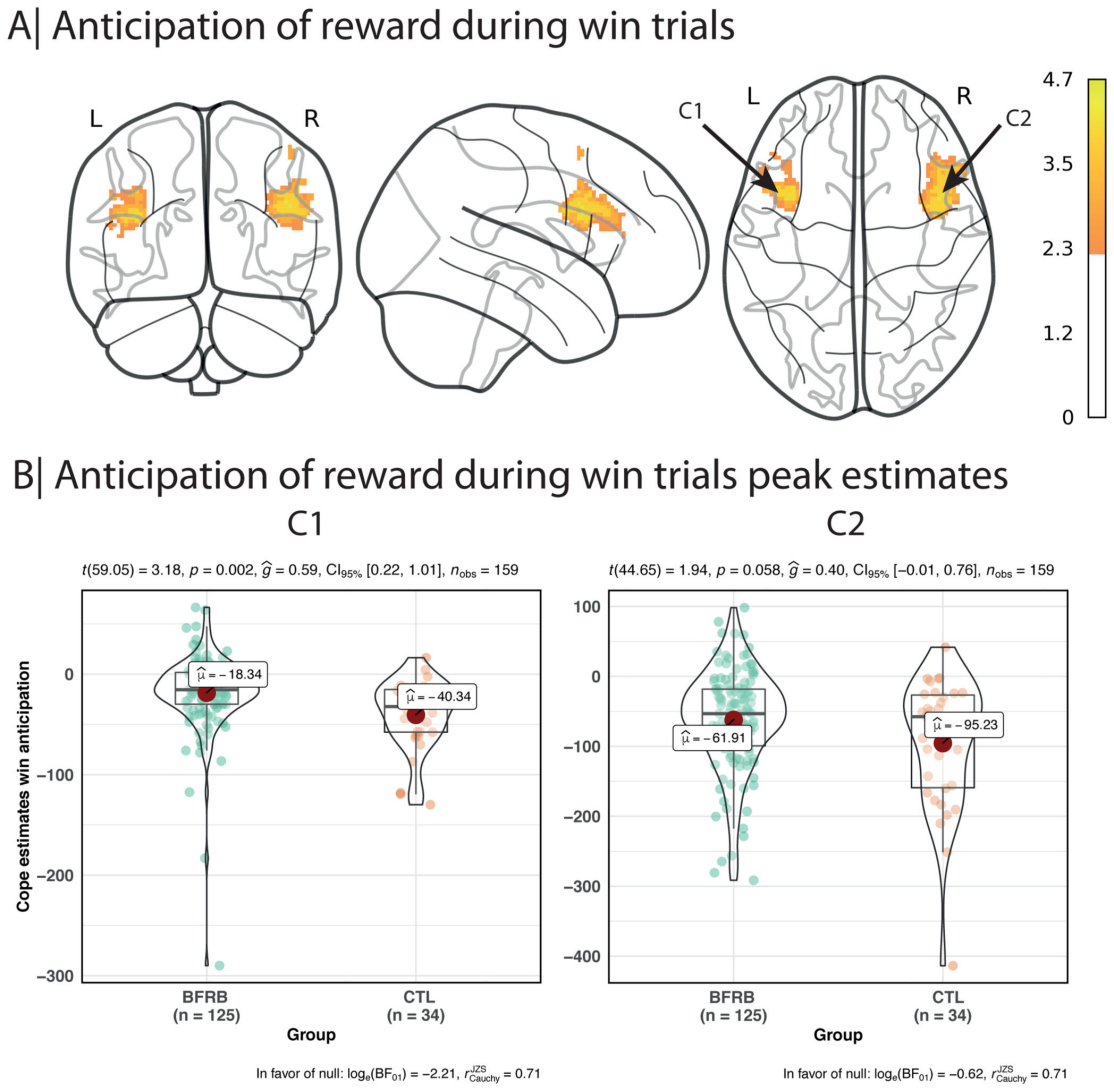
Anticipation of reward during win trials

Compared to controls, BFRB participants showed an increased activation in two significant clusters during the anticipation of reward (see Table 1 and Figure 1). Cluster 1 was maximal at left inferior frontal gyrus (BA 44 & 45). Cluster 2 was maximal at right inferior frontal gyrus (BA 44 & 45).

**Figure 2:**
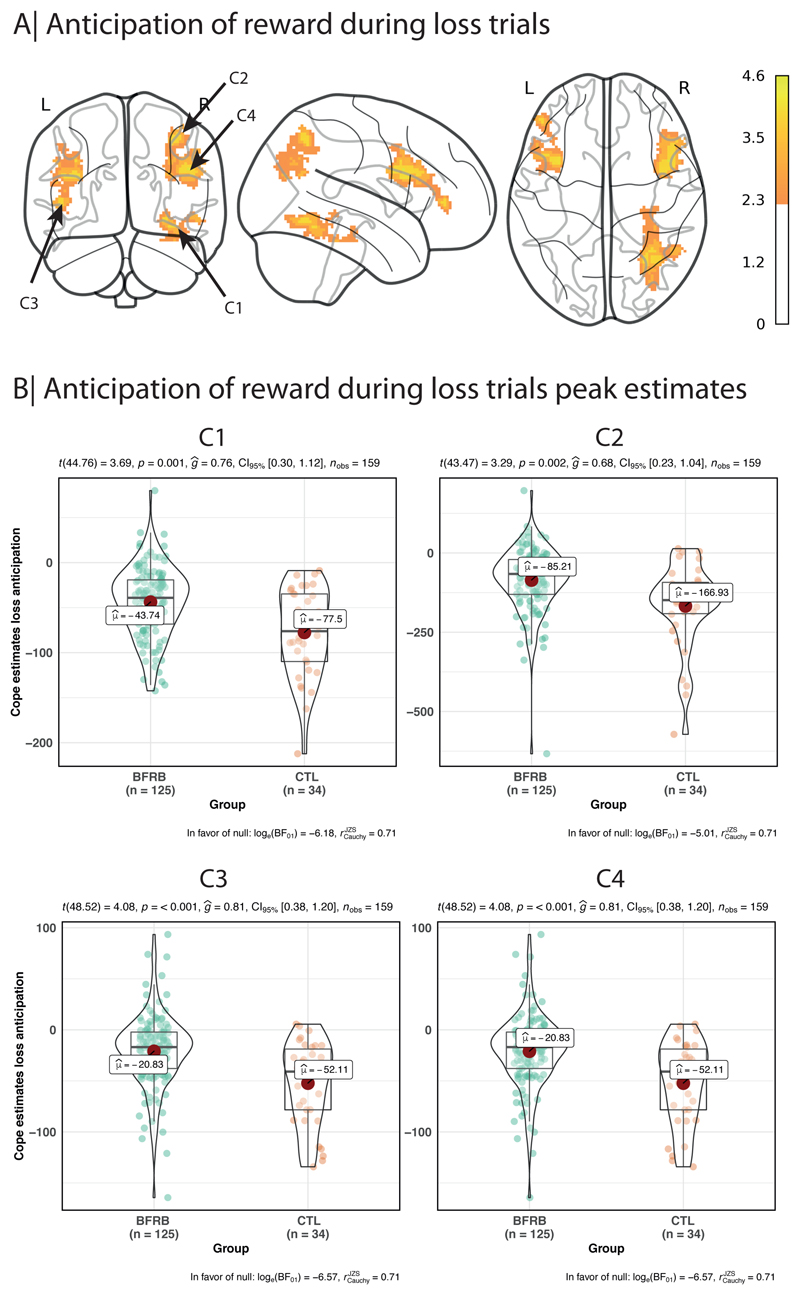
Anticipation of reward during loss trials

BFRB participants showed an increased activation in four significant clusters during the anticipation of loss (see Table 2 and Figure 2). Cluster 1 was maximal at right fusiform gyrus (BA 37), extending into inferior temporal gyrus, parahippocampal gyrus, and cerebellum. Cluster 2 was maximal at right inferior parietal lobule (BA 39), extending into angular gyrus and middle occipital gyrus. Cluster 3 was maximal at left inferior frontal gyrus (BA 44 & 45), extending into precentral gyrus and middle frontal gyrus. Cluster 4 was maximal at right inferior frontal gyrus (BA 44 & 45).

The BFRB and control groups did not differ significantly in the win-lose contrast. Additionally, activation at the peak cluster coordinates did not differ significantly between patients who did versus did not have other mental health comorbidities (all p>0.15 uncorrected, Wilcoxon tests).

**Figure 3:**
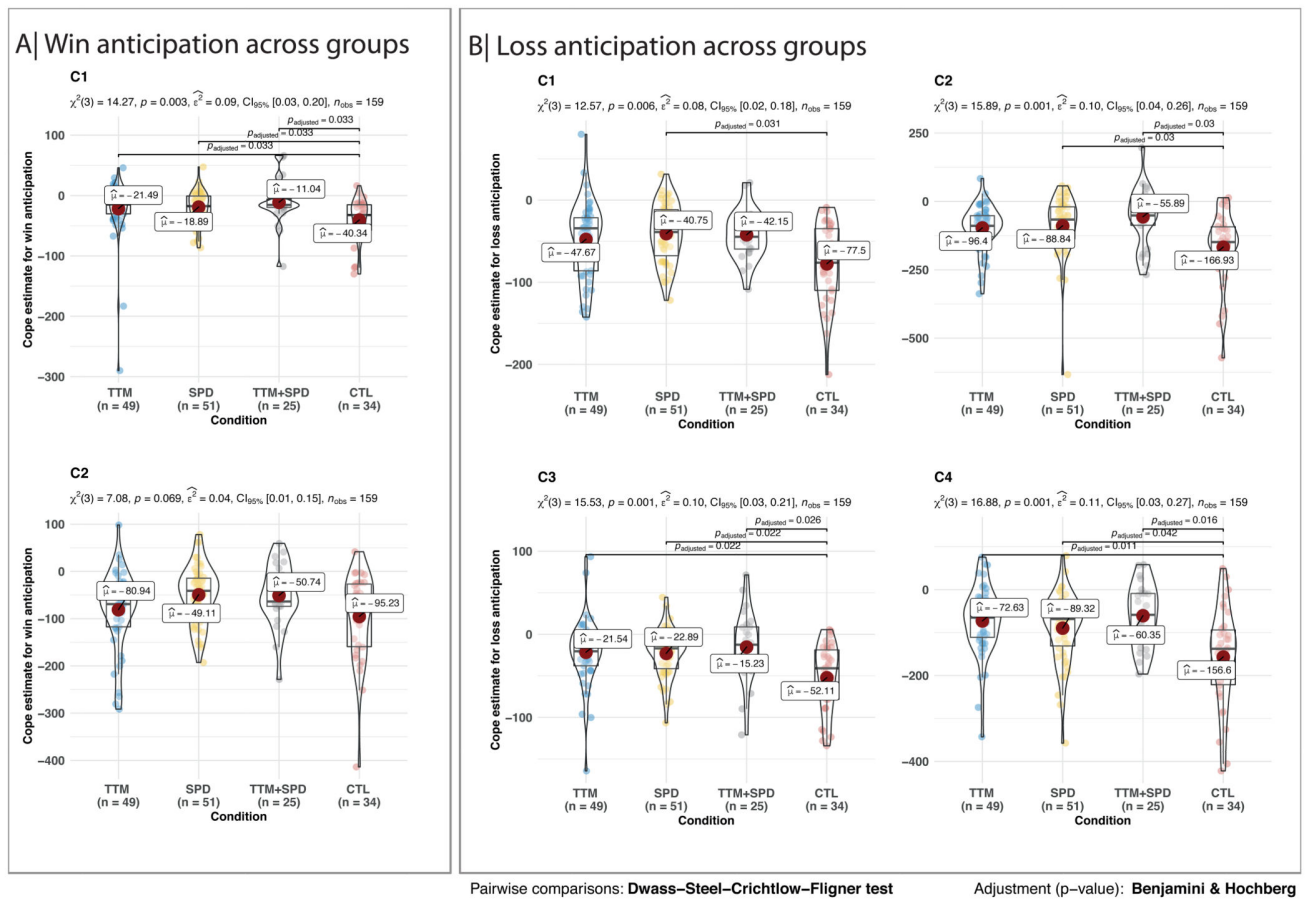
Win and loss anticipation across groups

After recognizing that BFRB participants differed from controls, we examined the groups within the BFRBs (TTM, SPD, and co-occurring TTM + SPD). Post-hoc analyses within condition are presented in Figure 3. Although the 3 clinical groups did not differ from each other on activation during anticipation of wins, all three clinical groups differed significantly from controls in Cluster 1 during anticipation of wins.

In terms of loss anticipation, all three clinical groups exhibited significantly greater activation in Clusters 3 and 4 compared to controls. In addition, participants with SPD exhibited significantly greater activation in Cluster 1 compared to controls. Finally, participants with SPD and those with SPD+TTM showed significantly greater activation in Cluster 2 compared to controls.

## Discussion

To our knowledge, this is the largest neuroimaging study of BFRBs to explore reward-related task activation. Our findings present evidence for dysregulated reward circuitry in BFRBs. Reward seeking and loss/harm avoidance play important roles in human behavior, and when there is dysfunction in reward processing, maladaptive behaviors may result. The DSM-5 diagnostic criteria for both TTM and SPD highlight failed attempts to reduce the behavior – which is suggestive of reward dysfunction. Here, the main finding was of marked hyperactivation of the bilateral inferior frontal gyrus (pars opercularis and pars triangularis) in people with BFRBs compared to controls, when anticipating reward or punishment. These results were significant with stringent statistical correction, including when controlling for potential confounders.

The various versions of the monetary incentive task have been useful in understanding the neuropathology underpinning reward processing. Though earlier work with this task focused on the nucleus accumbens (NAcc) using a region-of-interest approach, is now widely established that reward and loss anticipation involve activation of distributed neural circuitry. One neuroimaging meta-analysis (using 20 studies) showed that healthy volunteers activate the nucleus accumbens, thalamus, insula, and medial frontal gyrus during reward processing (Knutson and Greer 2008). A larger meta-analysis of 142 studies found that healthy volunteers activated the nucleus accumbens, insula, inferior frontal gyrus, anterior cingulate cortex, and medial orbito-frontal cortex during reward processing (Liu et al., 2011).

In terms of anticipation of rewards, we found strong evidence of hyperactivation in the inferior frontal gyrus (IFG) (as well as right fusiform gyrus, inferior temporal gyrus, parahippocampal gyrus, right inferior parietal lobule, and middle frontal gyrus) in all three BFRB groups compared to controls and these findings suggest that people with BFRBs are biologically hypersensitive to potential rewards. The salient role of the IFG is of particular interest given that above-mentioned meta-analysis found IFG activation (and the other regions) is common in reward processing tasks including during reward anticipation (Liu et al., 2011). Additionally, a small study of cognitive flexibility in TTM (n=12) found right frontal hyperactivation using fMRI (Grant et al., 2018) and excess IFG thickness was found in a recent meta-analysis in TTM (n=76 patients versus n=41 controls) (Wagnegger and Schienle 2019), which also seems to extend to first-degree relatives in a small sample (Odlaug et al., 2014). These findings collectively point to abnormalities of IFG as a potential core feature of BFRB, and now extends those previous TTM findings to SPD as well.

In addition to its role in reward and punishment anticipation noted above, the IFG is involved in the detection of environmentally salient cues (Hampshire et al., 2010), and in the suppression of habitual response patterns (Aron et al., 2014). Response inhibition deficits have been found in individuals with right IFG damage, and the severity of the response inhibition deficit has been positively associated with the degree of right IFG damage (Aron et al., 2003). Additionally, research has demonstrated that when the right IFG is disrupted by transcranial magnetic stimulation, response inhibition is also impaired (Chambers et al., 2007). The finding of abnormal IFG function in BFRBs may help to account for neuropsychological findings with regards to these disorders. In particular, significant inhibitory control deficits have been reported in most but not all studies of BFRBs versus healthy controls. One interpretation of the current data alongside the neurocognitive literature is that the IFG may be over-activated by anticipation of reward or punishment in patients with BFRBs, in turn impeding the ability of this region to successfully undertake other cognitive functions such as top-down behavioral inhibition of automated behavior. This hypothesis could be tested in future work by also examining the monetary incentive task and stop-signal fMRI task performance in relation not only to brain activation but also connectivity metrics. If this hypothesis is correct, treatment of BFRBs using medications or psychotherapies that dampen the sub-cortical reward pathways may then in turn enable the IFG to exert more top-down control by freeing up this region’s processing capacity.

Our results confirm an association between the anticipation of monetary reward and frontal hyperactivation. Having said that, the lack of a difference in striatal activation between BFRBs and controls merits further consideration. Unlike a recent fMRI study that found some evidence for abnormal accumbens activation in TTM patients during a reward task (White et al., 2013), our study found that activation of the accumbens did not differ between BFRB participants and controls. The task did activate this region across all subjects, indicating this null result was not simply due to the task failing to activate this region. The study by White and colleagues included only 13 subjects, all of whom had TTM, and thus differs from the current study in multiple ways. First, the accumbens finding in the previous study was not significant in the whole-brain analysis and so may reflect a false positive with respect to the region-of-interest analysis. Second, the role of the striatum in BFRBs is not well understood, with some previous structural studies finding that neither TTM nor SPD subjects differed significantly from controls in terms of dorsal and ventral striatum volumes (Odlaug et al., 2014) and others finding significant differences (Roos et al., 2015). A recent meta-analysis of all available literature did not find sub-cortical structural differences in TTM versus controls, including specifically in the NAcc (Chamberlain et al., 2018). Third, tasks that recruit both executive and reward networks may simply exhibit greater dyfunction in top-control elements of reward compared to bottom-up drive. Findings of no differences in NAcc activation during anticipated monetary reward seem to differ from those found in OCD (reduced NAcc activation to anticipated monetary reward in OCD (Figee et al., 2011) or those found in substance addictions (relatively increased NAcc response to rewarding outcome in cocaine addicted adults (Jia et al., 2011) and alcoholics (Bjork et al., 2008) and might suggest that BFRBs have distinct neurobiological substrates from these other conditions. Lastly, it should be noted that due to its small size, the NAcc can be considered difficult to visualize and measure in terms of activation, which could hinder ability to detect subtle differences in activation.

Several limitations should be considered in relation to the current study. The study was neither designed nor powered to evaluate possible effects of previous treatment on brain activation, nor the contribution of specific types of comorbidities. Overall though, brain findings did not differ as a function of whether patients did or did not have mental health comorbidities. Also, although the total size of the study was fairly large, the number of participants with individual BFRBs may have been too small to detect differences between those with TTM, SPD or the comorbid condition. Finally, the current research was undertaken in a cohort that was largely female and of white racial-ethnic type and thus may not be representative of the larger population of people with BFRBs.

In summary, this large multi-center fMRI study suggests that TTM and SPD are associated with disordered reward processing, linked to the inferior frontal gyrus. Ideally, understanding reward processing dysfunction should allow for improved treatment strategies via neuromodulation, pharmacotherapy or psychosocial interventions.

## Figures and Tables

**Table 1:** Cluster sizes and p values, along with peak coordinates for anticipation of reward hyperactivation in the BFRB group

Cluster Index	Voxels	P-corrected	Z-MAX	Z-MAX X (mm)	Z-MAX Y (mm)	Z-MAX Z (mm)
2	879	0.00102	4.14	44	10	30
1	481	0.048	4.19	-38	10	22

**Table 2:** Cluster sizes and p values, along with peak coordinates for anticipation of loss hyperactivation in the BFRB group.

Cluster Index	Voxels	P-corrected	Z-MAX	Z-MAX X (mm)	Z-MAX Y (mm)	Z-MAX Z (mm)
4	816	0.00162	4.22	46	12	28
3	729	0.00365	4.08	-36	12	24
2	537	0.0248	3.98	38	-60	50
1	506	0.0343	4.16	30	-60	-10

## Data Availability

Data available upon request

## Code availability

NA
